# Associations Between Depressive Symptoms, Cognitive Impairment, and Work Productivity Loss in Patients With Bipolar Disorder: A 48‐Week Longitudinal Analysis

**DOI:** 10.1002/npr2.70036

**Published:** 2025-08-04

**Authors:** Yoshikazu Takaesu, Ayano Shiroma, Tadashi Nosaka

**Affiliations:** ^1^ Department of Neuropsychiatry, Graduate School of Medicine University of the Ryukyus Okinawa Japan; ^2^ Medical Affairs Sumitomo Pharma co., Ltd. Tokyo Japan

**Keywords:** bipolar disorder, cognitive dysfunction, depression, presenteeism, quality of life

## Abstract

**Aim:**

To examine the relationships among work productivity loss and changes in depressive symptoms and cognitive impairment in patients with bipolar disorder and to investigate the relationship between bipolar disorder symptoms and work productivity loss.

**Methods:**

This prospective, 48‐week, longitudinal, web‐based, cohort questionnaire study included adults with bipolar disorder in Japan who were employed or on sick leave. Questionnaire surveys including validated self‐administered rating scales assessing cognitive impairment, work productivity loss, quality of life (QOL), depressive symptom severity, and sleep disturbance were completed every 12 weeks from baseline through Week 48. The primary endpoint was the correlation between change from baseline at 48 weeks in cognitive impairment and work productivity loss. Secondary endpoints included change from baseline in each symptom score, cognitive impairment, work productivity loss, depressive symptoms, QOL, and sleep disturbance.

**Results:**

Of 211 study participants, 179 responded to all the study questionnaires and were included in this 48‐week analysis. There was a weak correlation between change in cognitive impairment and change in work productivity loss (presenteeism) from baseline to Week 48 (Pearson correlation coefficient = 0.304, *p* = 0.001), but the multiple regression analysis showed no association. Change in work productivity loss was significantly associated with change in depressive symptoms (regression coefficient = 2.43, *p* < 0.001). Change in QOL was significantly associated with change in insomnia (regression coefficient = −0.01, *p* < 0.05) in multiple regression analyses.

**Conclusion:**

Treating patients with bipolar disorder to improve depressive symptoms and cognitive impairment may benefit work productivity.

## Abstract 2

This was a prospective, 48‐week, longitudinal, web‐based, cohort questionnaire study that revealed correlations between cognitive impairment/depressive symptoms and work productivity loss in patients with bipolar disorder who were employed or on sick leave in Japan. Treatment focusing on depressive symptoms and cognitive impairment may benefit work productivity.

## Graphical abstract

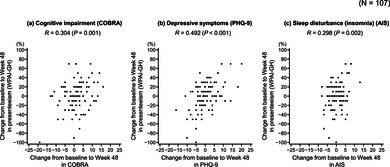

## 1 Introduction

Bipolar disorder is a type of mood disorder characterized by recurrent manic and depressive episodes [1]. Bipolar disorder has two types, Type I and Type II, depending on whether the manic episodes are hypomanic, and both types experience depressive episodes [2]. Long‐term prospective studies have found that depressive episodes are longer in duration than manic episodes, which highlights the importance of targeting the depressive phase of the disorder for treatment [3, 4].

The core symptoms of patients with bipolar disorder include loss of satisfaction, pleasure, and interest, and symptoms of cognitive impairment [5, 6, 7, 8], sleep disturbances [9, 10], and anxiety [11]. Depressive symptoms and cognitive deficits in patients with bipolar disorder are positively associated with poor occupational functioning [12]. Patients with bipolar disorder may experience cognitive impairment even in the euthymic state, which is associated with reduced quality of life (QOL) [13, 14, 15] and social functioning [7, 16, 17, 18]. One study showed that patients with bipolar disorder had greater work productivity loss than adults who had never had bipolar disorder, schizophrenia, or major depressive disorder [19]. A cross‐sectional assessment using the Cognitive Complaints in Bipolar Disorder Rating Assessment (COBRA) found that in general adult workers in Japan, there was an association between cognitive complaints and work productivity loss [20]. To date, there have been no longitudinal studies on the relationship between cognitive impairment and work productivity loss in patients with bipolar disorder, and this relationship remains to be clarified.

Given the evidence gap, we conducted a longitudinal study evaluating the relationship between changes in cognitive impairment and changes in work productivity loss in patients with bipolar disorder who attended a medical institution and worked (employed or on sick leave) during the 48‐week observation period. The baseline, cross‐sectional analysis of this study [21] found that depressive symptoms, cognitive impairment, and insomnia sleep disturbances were associated with work productivity loss and reduced QOL, which is consistent with a previous study of patients with bipolar disorder [19].

We report here the 48‐week follow‐up findings of the abovementioned study, which included a longitudinal questionnaire survey that aimed to prospectively assess work productivity loss, employment status, and medical treatment status. Our goal was to examine the relationships among these factors, including work productivity loss and changes in cognitive impairment, and QOL in patients with bipolar disorder. The relationship between depressive symptoms and both work productivity loss and QOL was also assessed.

## 2 Methods

### 2.1 Study Design

This was a prospective, 48‐week, longitudinal, web‐based, cohort questionnaire study of patients with bipolar disorder conducted in Japan between July 2023 and September 2024. Details of the study design and the initial cross‐sectional analysis were previously published [21]. The questionnaire survey was conducted and analyzed by QLife Inc. Tokyo, Japan. Participants were to complete questionnaires every 12 weeks.

The study protocol was approved by the University of the Ryukyus Ethics Review Committee for Life Science and Medical Research Involving Human Subjects. This research complies with the ethical principles and relevant notifications stipulated in the Declaration of Helsinki (as revised in 2013), and the study was registered in UMIN‐CTR under the identifier UMIN000051519. All participants provided electronic informed consent to participate.

### 2.2 Study Participants

Briefly, the study included patients from the QLife web system who were being treated for bipolar disorder at a medical institution at the time of the first questionnaire assessment, were aged 18–59 years at the time of informed consent, were employed or on sick leave, and were able to complete the web questionnaire. Patients who were hospitalized or had a diagnosis of dementia at baseline, as well as those who did not plan to return to work or find employment in the next 12 months, were excluded. Additional details of the inclusion and exclusion criteria can be found in the initial cross‐sectional analysis publication [21].

### 2.3 Outcome Measures

Cognitive impairment was assessed using the COBRA rating scale [22, 23], from which a total score for each patient was calculated. The Work Productivity and Activity Impairment Questionnaire: General Health (WPAI‐GH) [24, 25] was used to evaluate presenteeism, absenteeism, overall work impairment, and activity impairment. Medical resource use and employment status were assessed from the number of hospital visits, hospitalizations, and days in hospital, as well as hospitalization rates and rates of absence from work and return to work. The Health Utilities Index Mark 3 (HUI3) was used to assess QOL [26, 27] and to calculate a total utility value (scores of 1.00 indicate perfect health). Depressive symptom severity was assessed using the Patient Health Questionnaire‐9 (PHQ‐9) [28, 29], and the total depressive symptom severity score was calculated. Sleep disturbance was assessed using the Epworth Sleepiness Scale (ESS) [30, 31] and the Athens Insomnia Scale (AIS) [32, 33]; the total score was calculated for each.

### 2.4 Study Endpoints

The primary endpoint was the correlation between change from baseline at 48 weeks in cognitive impairment and work productivity loss (presenteeism, absenteeism, overall work impairment, and activity impairment). The secondary endpoints included change from baseline in each assessment scale score: cognitive impairment, depressive symptoms, sleep disturbance (ESS and AIS), work productivity loss (presenteeism, absenteeism, overall work impairment, activity impairment), and QOL (HUI3). Other secondary endpoints included relationships of change from baseline in symptom scores; correlation of work productivity loss with symptom scores; factors associated with change from baseline at 48 weeks in work productivity loss and QOL; change in patient background or symptoms from baseline to 48 weeks; percent transition of depressive symptoms from baseline to each visit; and percentage of overall time spent with depressive symptoms (PHQ‐9 ≥ 10) during the observation period.

### 2.5 Statistical Analysis

The analyses in the present study used the per‐protocol set, which was defined as all participants who met the eligibility criteria, whose questionnaire responses were valid, and who responded to the questionnaires at all evaluation time points.

For each assessment measure (COBRA, PHQ‐9, ESS, and AIS), the cutoff scores for the absence or presence of each symptom were as follows: COBRA ≤ 14 versus > 14 (cognitive impairment) [34, 35], PHQ‐9 < 10 versus ≥ 10 (depressive symptoms) [28, 29], ESS ≤ 10 versus > 10 (somnolence) [30, 36], AIS < 10 versus ≥ 10 (insomnia) [37].

Correlations of each symptom (cognitive impairment, depressive symptoms, and insomnia) with work productivity loss and QOL (HUI3) were determined by creating scatter plots and calculating Pearson correlation coefficients. Indirect cost was calculated as described in Takaesu et al. (Text S1) [21].

Single and multiple regression analyses were conducted with change from baseline in work productivity loss (WPAI‐GH) and QOL (HUI3) as the objective variables and participant characteristics and each symptom as explanatory variables. In the multiple regression analysis, explanatory variables were selected based on multiple aspects: results of single regression analysis, clinical perspective, appropriate number of explanatory variables based on the study population number, and avoidance of multicollinearity.

Missing values were not imputed for survey items. The statistical analyses were performed using SAS version 9.4 software (SAS Institute Inc., Cary, NC, USA). All statistical tests were two‐tailed and had a 5% level of significance, with no adjustment for multiple comparisons.

## 3 Results

### 3.1 Participant Characteristics

As previously reported, of the 353 participants with bipolar disorder who provided electronic informed consent and were enrolled in the study, 211 were included in the analysis population of the overall study [21]. The 48‐week longitudinal analysis included 179 participants who responded to all study questionnaires for up to 48 weeks (i.e., the study duration).

Baseline participant characteristics have been previously reported [21]. Briefly, the mean age was 38.9 years, 64.9% were female, 41.2% were working full time, and 40.3% had comorbidities. The mean current employment duration was 4.6 years, and the mean duration of bipolar disorder was 7.2 years. Cognitive impairment (COBRA > 14) was present in 59.7% of participants, and depressive symptoms (PHQ‐9 ≥ 10) were present in 67.8% of participants. The mean WPAI‐GH presenteeism was 45.1%, the mean indirect cost was 1688.0 thousand Japanese yen, and the mean QOL utility value (HUI3) was 0.461. Changes in participant characteristics from baseline through Week 48 are shown in Table S1. At Week 48, 37.1% of participants were working full time, and 17.4% were on sick leave. Cognitive impairment (COBRA > 14) was present in 65.7% of participants, and there was no significant change in the COBRA mean score from baseline to 48 weeks. Depressive symptoms (PHQ‐9 ≥ 10) were present in 61.8% of participants, and there was no significant change in the PHQ‐9 mean score from baseline to 48 weeks. The proportion of patients with depressive symptoms both at baseline and at 48 weeks was 52.0% (93/179), and the proportion of patients without depressive symptoms both at baseline and at 48 weeks was 22.9% (41/179) (Table S2). Of the 120 patients with depressive symptoms at baseline, 93 (77.5%) patients still had depressive symptoms at 48 weeks (Table S2). On the other hand, 15.1% of the respondents reported an improvement change from baseline to 48 weeks, while 10.1% reported a worsening change. The mean percentage (mean ± SD) of time spent with depressive symptoms (PHQ‐9 ≥ 10) during the entire observation period was 65.4 ± 40.0%. The mean WPAI‐GH presenteeism was 43.7%, the mean indirect cost was 1620.6 thousand Japanese yen, and the mean QOL utility value (HUI3) was 0.466.

### 3.2 Correlations Between Symptoms

**FIGURE 1:**
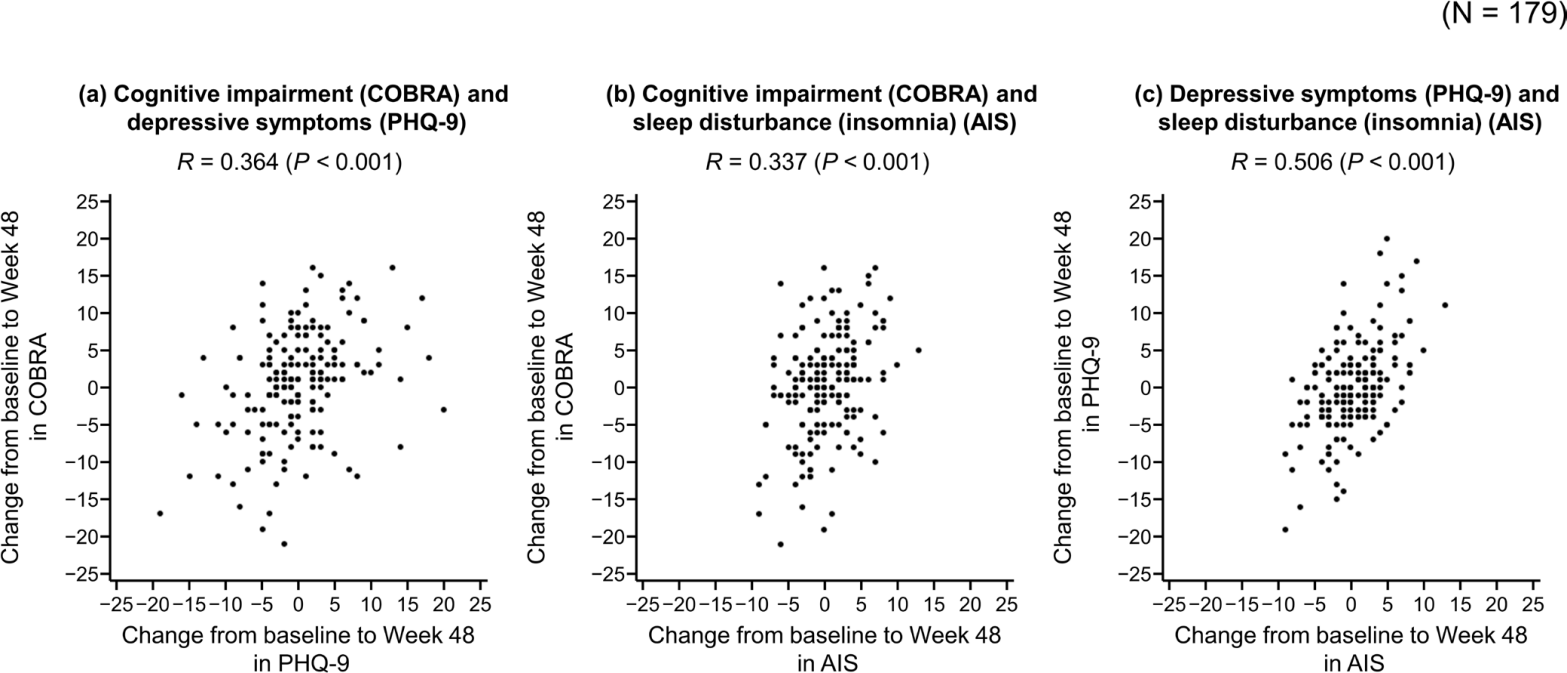
Scatter plots showing the correlations between change from baseline to Week 48 in cognitive impairment (COBRA) and depressive symptoms (PHQ‐9) (a), cognitive impairment (COBRA) and sleep disturbance (insomnia) (AIS) (b), and depressive symptoms (PHQ‐9) and sleep disturbance (insomnia) (AIS) (c). AIS, Athens Insomnia Scale; COBRA, Cognitive Complaints in Bipolar Disorder Rating Assessment; PHQ‐9, Patient Health Questionnaire‐9; *R*, Pearson correlation coefficient.

At 48 weeks, change from baseline in cognitive impairment (COBRA) was weakly correlated with change from baseline in depressive symptoms (PHQ‐9) (Pearson correlation coefficient [*R*] = 0.364, *p* < 0.001) (Figure 1a) and insomnia (AIS) (*R* = 0.337, *p* < 0.001) (Figure 1b). A moderate correlation between change from baseline in depressive symptoms (PHQ‐9) and insomnia (AIS) was also observed (*R* = 0.506, *p* < 0.001) (Figure 1c). Correlations between these symptoms at Weeks 12, 24, and 36 are shown in Table S3.

### 3.3 Work Productivity Loss

**FIGURE 2:**
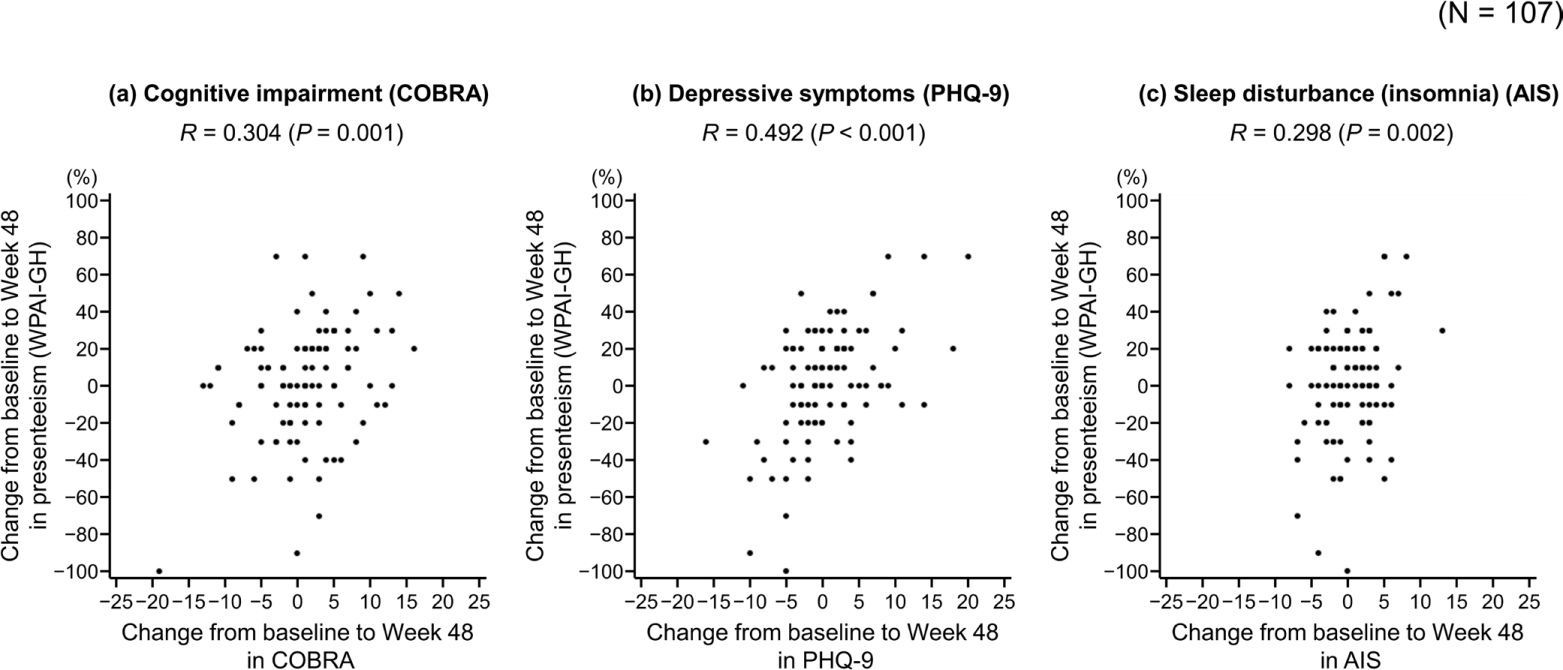
Scatter plots showing the correlations between change from baseline to Week 48 in work productivity loss (presenteeism) and cognitive impairment (COBRA) (a), depressive symptoms (PHQ‐9) (b), and sleep disturbance (insomnia) (AIS) (c). AIS, Athens Insomnia Scale; COBRA, Cognitive Complaints in Bipolar Disorder Rating Assessment; PHQ‐9, Patient Health Questionnaire‐9; *R*, Pearson correlation coefficient; WPAI‐GH, Work Productivity and Activity Impairment Questionnaire‐General Health.

For the primary endpoint, there was a weak correlation between change from baseline to 48 weeks in cognitive impairment (COBRA) and work productivity loss (presenteeism) (*R* = 0.304, *p* = 0.001) (Figure 2a). Similarly, a significant correlation was observed between cognitive impairment and both overall work impairment (*R* = 0.264, *p* = 0.006) and activity impairment (*R* = 0.195, *p* = 0.022), but not between cognitive impairment and absenteeism (*R* = 0.095, *p* = 0.310) (Table S4). The changes from baseline to Week 48 in both depressive symptoms (PHQ‐9) and insomnia (AIS) were significantly correlated with work productivity loss (presenteeism) (*R* = 0.492, *p* < 0.001 and *R* = 0.298, *p* = 0.002, respectively) (Figure 2b,c).

The full results of single and multiple regression analyses for variables associated with work productivity loss (presenteeism, absenteeism, overall work impairment, and activity impairment) are shown in Tables S5 and S6, respectively. The single regression analysis showed that duration of employment (presenteeism), change in cognitive impairment (presenteeism, overall work impairment, and activity impairment), change in depressive symptoms (presenteeism, absenteeism, overall work impairment, and activity impairment), and change in sleep disturbance (insomnia) (presenteeism, absenteeism, overall work impairment, and activity impairment) were significantly associated with work productivity loss. The multiple regression analysis showed that duration of current employment and change in depressive symptoms (PHQ‐9) were significantly associated with work productivity loss (presenteeism) (regression coefficient = −0.81, *p* = 0.013 and 2.43, *p* < 0.001, respectively) (Table 1).

**TABLE 1:** Multiple regression analysis: Change from baseline to Week 48 in work productivity loss (presenteeism).

		*R* ^2^ = 0.302
Explanatory variable	Regression coefficient (95% CI)	*p*
Duration of current employment, years	−0.81 (−1.44, −0.18)	0.013
Change in cognitive impairment (COBRA)	0.68 (−0.16, 1.51)	0.111
Change in depressive symptoms (PHQ‐9)	2.43 (1.38, 3.47)	< 0.001
Change in sleep disturbance (insomnia) (AIS)	0.02 (−1.62, 1.66)	0.980

Abbreviations: AIS, Athens Insomnia Scale; CI, confidence interval; COBRA, Cognitive Complaints in Bipolar Disorder Rating Assessment; PHQ‐9, Patient Health Questionnaire‐9; *R* ^2^, coefficient of determination.

### 3.4 QOL

**FIGURE 3:**
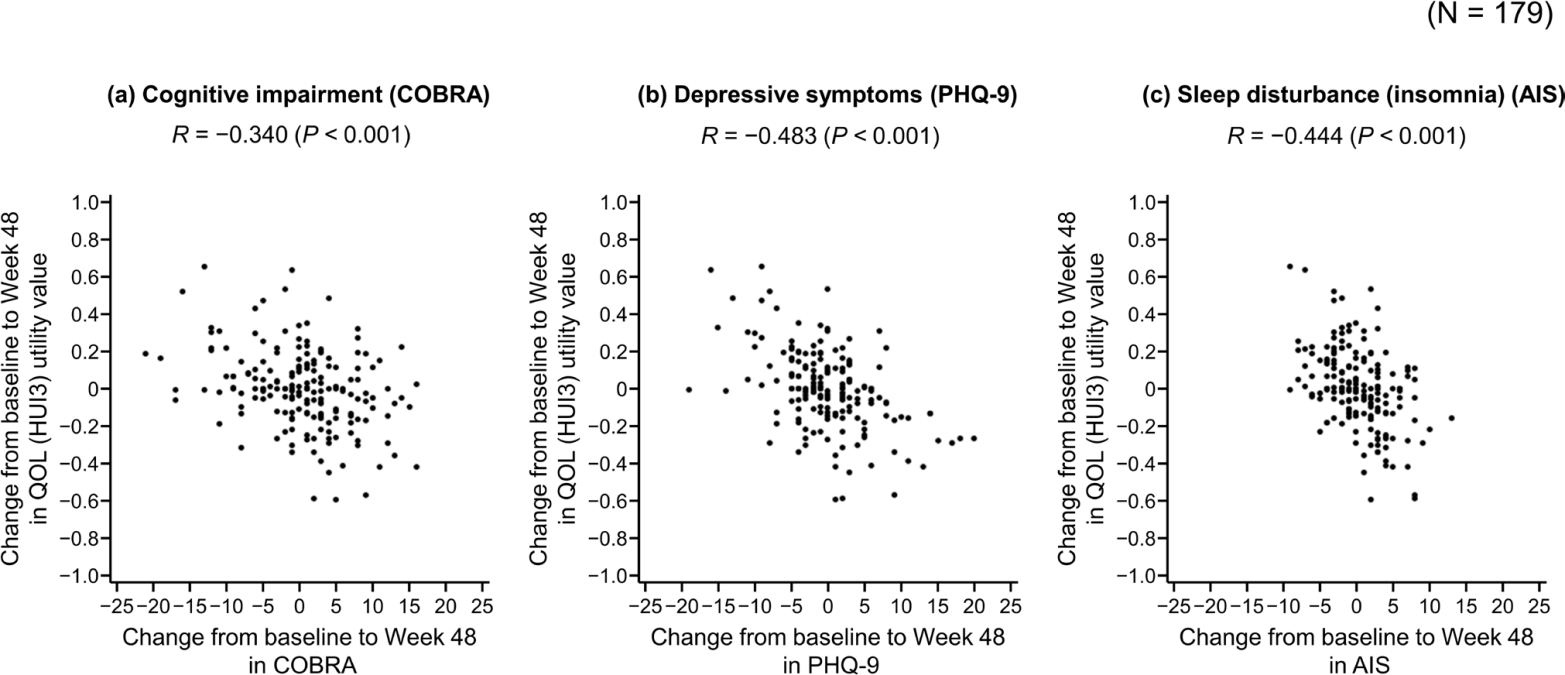
Scatter plots showing the correlations between change from baseline to Week 48 in QOL (HUI3) and cognitive impairment (COBRA) (a), depressive symptoms (PHQ‐9) (b), and sleep disturbance (insomnia) (AIS) (c). AIS, Athens Insomnia Scale; COBRA, Cognitive Complaints in Bipolar Disorder Rating Assessment; HUI3, Health Utilities Index Mark 3; PHQ‐9, Patient Health Questionnaire‐9; QOL, quality of life; *R*, Pearson correlation coefficient.

The change from baseline to Week 48 in QOL (HUI3 utility score) had weak negative correlations with cognitive impairment (*R* = −0.340, *p* < 0.001), depression (*R* = −0.483, *p* < 0.001), and insomnia (*R* = −0.444, *p* < 0.001) (Figure 3).

The full results of the single and multiple regression analyses for variables associated with QOL are shown in Table S7 and Table 2, respectively. In the single regression analysis, QOL was significantly associated with depressive symptoms, insomnia (AIS), alcohol use, change from baseline to Week 48 in cognitive impairment (COBRA), depressive symptoms (PHQ‐9), insomnia (AIS), and work productivity loss (presenteeism, absenteeism, overall work impairment, and activity impairment) (Table S7). In the multiple regression analysis, change from baseline to Week 48 in sleep disturbance (insomnia) (AIS) was significantly associated with QOL (HUI3) (regression coefficient = −0.01, *p* < 0.05) (Table 2).

**TABLE 2:** Multiple regression analysis: Change from baseline to Week 48 in QOL (HUI3).

		*R* ^2^ = 0.222
Explanatory variable	Regression coefficient (95% CI)	*p*
Change in cognitive impairment (COBRA)	−0.01 (−0.01, 0.00)	0.099
Change in depressive symptoms (PHQ‐9)	−0.01 (−0.02, 0.00)	0.108
Change in sleep disturbance (insomnia) (AIS)	−0.01 (−0.03, 0.00)	< 0.05
Change in WPAI‐GH (presenteeism)	−0.00 (−0.00, 0.00)	0.425
Change in WPAI‐GH (absenteeism)	0.00 (−0.00, 0.00)	0.781

Abbreviations: AIS, Athens Insomnia Scale; CI, confidence interval; COBRA, Cognitive Complaints in Bipolar Disorder Rating Assessment; ESS, Epworth Sleepiness Scale; HUI3, Health Utilities Index Mark 3; PHQ‐9, Patient Health Questionnaire‐9; QOL, quality of life; *R* ^2^, coefficient of determination; WPAI‐GH, Work Productivity and Activity Impairment Questionnaire‐General Health.

There was no multicollinearity between explanatory variables (variance inflation factor, < 10) in all multiple regression analyses presented.

## 4 Discussion

To the best of our knowledge, this is the first study to longitudinally evaluate changes in cognitive impairment and work productivity loss over a 48‐week period in employed patients receiving treatment for bipolar disorder. A weak but significant positive correlation was found between change in cognitive impairment and change in work productivity loss (presenteeism, overall work impairment, and activity impairment) from baseline to 48 weeks, which was the primary endpoint. Similarly, using single regression analysis, change in cognitive impairment was found to be associated with change in work productivity loss. However, using a multiple regression analysis that adjusted for other confounding factors, change in cognitive impairment was not associated with change in work productivity loss.

In 1 to 5‐year follow‐up studies of patients with bipolar disorder who were in remission, cognitive function was found to affect occupational skills [12, 17, 38, 39]; however, these studies did not measure work productivity loss. Given the prospect of returning to work or continued employment, cognitive impairment may be an important target of treatment. In the present study, while a weak correlation was observed between cognitive impairment and work productivity loss, no statistically significant association was observed between these two variables in the multiple regression analysis. A possible reason for this finding is that 68% of patients had depressive symptoms when they entered the study even though the study included patients who were employed. Furthermore, the proportion of patients with depressive symptoms at 48 weeks was 62.0%, and the proportion of patients with depressive symptoms at baseline and 48 weeks was 77.5%, with no significant change in depressive symptom scale scores. While there may have been some changes, such as an improvement or worsening of depressive symptoms, for 48 weeks, it is likely that the majority of patients had no change in symptoms for an extended period. In clinical practice, physicians first treat patients with bipolar disorder in the acute phase with the aim of achieving symptomatic remission. Subsequently, they try to treat patients for functional remission with the goal of improving their life in society. In this study, the target population was assumed to be patients with bipolar disorder who were working and had achieved at least symptomatic remission. However, many of the participants were working with residual symptoms before cognitive impairment had improved. Furthermore, only 15.1% were depressed at baseline and not depressed at Week 48, making it impossible to assess the association between cognitive impairment and work productivity loss in this population with improved depression. Although changes in cognitive impairment were not associated with work productivity loss when assessed using the multiple regression analysis, correlation analysis and single regression analysis showed a correlation. Given that there was some degree of relationship between change in cognitive impairment and change in work productivity loss, it is advisable to consider cognitive impairment in the daily care of patients with bipolar disorder who are receiving treatment in the clinic while continuing to work.

Depressive symptoms were the only significant variable associated with work productivity loss in the multiple regression analysis. A 2‐year study evaluating the association between symptoms and work productivity loss in patients with bipolar disorder found that those with depressive symptoms experienced absenteeism approximately 4 days per month more than those without depressive symptoms [40]. This indicates that the severity of depressive symptoms affects work productivity loss (absenteeism). In the 1 to 5‐year follow‐up studies of patients with bipolar disorder in remission, cognitive function was found to affect occupational skills [12, 17, 36, 37]. In the present longitudinal analysis, changes in depressive symptoms affected changes in work productivity loss such as presenteeism, which is consistent with the aforementioned studies, suggesting that controlling core depressive symptoms is important for addressing work productivity loss such as presenteeism. It is also important to provide appropriate treatment for depressive symptoms, the core symptom of bipolar disorder, by referring to treatment guidelines [41].

In a cross‐sectional analysis conducted as part of the present study at baseline, approximately 68% of patients had depressive symptoms and 60% had cognitive impairment. At 48 weeks, the proportions of patients with these symptoms were approximately 62% and 66%, respectively. These scores did not significantly change over time, with mean scores ranging from 12.7 to 12.6 for the PHQ‐9 and 17.3 to 17.7 for COBRA. Previous studies that longitudinally assessed occupational skills and depressive symptoms in patients with bipolar disorder reported improvements in depressive symptoms over time. One study reported a change in PHQ‐9 scores from 10.6 at baseline to 9.31 at 1 year and 4.95 at 5 years [12] and another reported a change in Montgomery–Asberg Depression Rating Scale scores from 26.5 at baseline to 13.3 at 12 months [38]. The improvement in depressive symptoms was associated with changes in occupational skills. In addition, a study evaluating the association between depressive symptom severity and employment status found that patients with bipolar disorder who experienced depressive symptoms were approximately 15% less likely to be employed than those in remission [40]. Another study evaluating the association between mood symptoms and employment status among full‐time employees over a 1‐year observation period reported that patients with depression were at a higher risk for loss of employment compared with those in remission (odds ratio, 2.16) [42]. Despite the fact that some people are able to work while having residual depressive symptoms, they remain at risk of employment loss. To increase the likelihood of continued employment, it is essential that depressive symptoms are improved to the point of remission at the time a person returns to work. Although the present study included outpatients with bipolar disorder, many patients did not experience an improvement in depressive symptoms, worked with residual symptoms, and continued to work for 48 weeks with no improvement in their residual symptoms and work productivity loss. We speculate that there may have been some patients who were not provided with adequately effective treatment. However, this could not be confirmed as treatment information was not collected in our study. In order to verify this assumption, research is needed to clarify the relationship between treatment status and outcomes such as work productivity loss, QOL, and treatment satisfaction. Physicians should make efforts to provide better treatment that is adequately effective for patients with bipolar disorder. At a minimum, the use of typical antipsychotics and antidepressants that have an unfavorable impact on remission in bipolar disorder should be avoided as much as possible, based on evidence and treatment guidelines [41, 43, 44]. Additionally, it may be necessary to include reevaluation of the need for concomitant administration of antidepressants or benzodiazepines, which can negatively affect cognitive function [45, 46]. The present longitudinal study highlights the fact that some patients with bipolar disorder continue to work, despite not having achieved symptomatic remission, meaning that these patients endure depressive symptoms and other cognitive impairments while working.

QOL was also assessed in relation to each symptom, and a significant negative correlation was found between change in cognitive impairment and change in QOL from baseline to 48 weeks. However, change in insomnia was the only symptom associated with QOL in the multiple regression analysis. It was hypothesized that insomnia needed to be improved in order to improve QOL in patients with bipolar disorder. In the cross‐sectional analysis at baseline of this study [21], in addition to insomnia, depressive symptoms and cognitive impairment were associated with lower QOL. It is unclear whether depressive symptoms and cognitive impairment were associated with lower QOL in the longitudinal analysis, but one possible reason may be that sleep disturbances are more easily identified as a problem in patients with bipolar disorder, which may be associated with lower QOL. Also, as with work productivity loss, neither depressive symptoms nor cognitive impairment changed significantly over the 48‐week period, suggesting that they were not associated with a decline in QOL.

This study had several limitations that necessitate careful interpretation of the results. Generalization of the study findings is limited because of selection bias, as the eligibility criteria limited the target population, and it is assumed that many of the patients who participated in the study had a particular interest in bipolar disorder. Given that each of the symptom surveys used a self‐administered rating scale and the survey answers were not objectively evaluated by physicians and raters, the results should be interpreted with caution. Manic symptoms, which are a core symptom of bipolar disorder, could not be assessed in this study as there is no validated Japanese version of a self‐administered rating scale for manic symptoms. The possibility that manic symptoms affected work productivity loss cannot be ruled out. Data were not available for some potentially important confounding factors affecting cognitive impairment, including current medications to treat bipolar disorder (e.g., antipsychotics, benzodiazepines, mood stabilizers), bipolar subtype (type 1 or 2), and comorbid neurodevelopmental disorders such as attention deficit hyperactivity disorder.

In conclusion, this 48‐week longitudinal analysis showed that there was a weak correlation between change from baseline at 48 weeks in cognitive impairment and work productivity loss. However, change in depressive symptoms (core symptoms of bipolar disorder) was associated with change in work productivity loss.

## Author Contributions

All authors contributed to the conception or design of this study, interpretation of data, and drafting of the manuscript, and have read and approved the final manuscript.

## Ethics Statement

The study was approved by the University of the Ryukyus Ethics Review Committee for Life Science and Medical Research Involving Human Subjects. This research complies with the Declaration of Helsinki and the Ethical Guidelines for Life Sciences and Medical Research Involving Human Subjects. University Hospital Medical Information Network (UMIN) Clinical Trials Registry: UMIN000051519.

## Consent

All participants provided electronic informed consent to participate.

## Conflicts of Interest

Y.T. has received research funding from Otsuka Pharmaceutical Co. Ltd., Meiji Seika Pharma Co. Ltd., and Eisai Co. Ltd.; lecture fees from Takeda Pharmaceutical Co. Ltd., Sumitomo Pharma Co. Ltd., Otsuka Pharmaceutical Co. Ltd., Mochida Pharmaceutical Co. Ltd., Lundbeck Japan K.K., Viatris Pharmaceuticals Japan G.K., Nobelpharma Co. Ltd., Meiji Seika Pharma Co. Ltd., Eisai Co. Ltd., MSD K.K., Daiichi Sankyo Co. Ltd., and Shionogi & Co. Ltd., outside the submitted work. A.S. had no competing interests. T.N. is a full‐time employee of Sumitomo Pharma Co. Ltd.

## Supporting information


**Table S1.** Participant characteristics at baseline through Week 48 and change from baseline at each time point.
**Table S2.** Percent transition of depressive symptoms from baseline to each visit (*N* = 179).
**Table S3.** Correlations between symptom scores.
**Table S4.** Correlation of work productivity loss with COBRA, PHQ‐9, and AIS.
**Table S5.** Single regression analysis: change from baseline to Week 48 in WPAI‐GH.
**Table S6.** Multiple regression analysis: change from baseline to Week 48 in WPAI‐GH.
**Table S7.** Single regression analysis: change from baseline to Week 48 in QOL (HUI3).

## Data Availability

The data from this study have not been made publicly available because the disclosure of individual data was not specified in the study protocol, and consent for public data sharing was not obtained from the participants.
