# Supplementary material for: Associations Between Depressive Symptoms, Cognitive Impairment, and Work Productivity Loss in Patients With Bipolar Disorder: A 48‐Week Longitudinal Analysis
Source: Neuropsychopharmacol Rep. 2025 Aug 4;45(3):e70036. doi: 10.1002/npr2.70036 (PMC12320119; doi:10.1002/npr2.70036)
Supplement: Supplementary file 1 — Table S1. Participant characteristics at baseline through Week 48 and change from baseline at each time point. Table S2. Percent transition of depressive symptoms from baseline to each visit (N = 179). Table S3. Correlations between symptom scores. Table S4. Correlation of work productivity loss with COBRA, PHQ‐9, and AIS. Table S5. Single regression analysis: change from baseline to Week 48 in WPAI‐GH. Table S6. Multiple regression analysis: change from baseline to Week 48 in WPAI‐GH. Table S7. Single regression analysis: change from baseline to Week 48 in QOL (HUI3). [file NPR2-45-e70036-s001.docx]

**Supporting information**

**Text S1.** Indirect cost calculation

Indirect cost was calculated using the Basic Survey on Wage Structure^1^ and the following formula: absenteeism cost = applicable monthly wage by sex and age × absenteeism ratio; presenteeism cost = applicable monthly wage by sex and age × presenteeism ratio; indirect cost = applicable monthly wage by sex, age × overall work impairment ratio (%) (for annual salary × 12 months) (as previously described in Takaesu et al.^2^).

**References**

1. Ministry of Health, Labour and Welfare. Wage Structure in the Basic Survey on Wage Structure, 2022 Edition. March 17, 2023. [Cited 24 January 2025.] Available from: https://www.mhlw.go.jp/toukei/itiran/roudou/chingin/kouzou/z2022/dl/13.pdf.
2. Takaesu Y, Shiroma A, Nosaka T, Maruyama H. Associations between cognitive impairment, depressive symptoms, and work productivity loss in patients with bipolar disorder: a cross-sectional analysis. Neuropsychopharmacol Rep. 2025 Mar; 45(1): e70012.

**Table S1** Participant characteristics at baseline through Week 48 and change from baseline at each timepoint

|  | **Baseline**  **(N = 178)** | **Week 12**  **(N = 178)** | **Week 24**  **(N = 178)** | **Week 36**  **(N = 178)** | **Week 48**  **(N = 178)** |
| --- | --- | --- | --- | --- | --- |
| Employment status |  |  |  |  |  |
| Working: full time | 71 (39.9) | - | - | - | 66 (37.1) |
| Working: part time | 46 (25.8) | - | - | - | 43 (24.2) |
| Working: self-employed | 10 (5.6) | - | - | - | 9 (5.1) |
| Working: continuous employment support | 14 (7.9) | - | - | - | 12 (6.7) |
| Transition support for employment | 8 (4.5) | - | - | - | 3 (1.7) |
| On sick leave | 29 (16.3) | - | - | - | 31 (17.4) |
| Living alone (single) | 49 (27.5) | - | - | - | 55 (30.9) |
| Not living alone (not single) | 129 (72.5) | - | - | - | 123 (69.1) |
| Non-national health insurance | 117 (65.7) | - | - | - | 120 (67.4) |
| National health insurance | 61 (34.3) | - | - | - | 58 (32.6) |
| Sleep duration (weekdays), hours/day | 7.4 ± 1.6 | - | - | - | 7.5 ± 1.8 |
| Sleep duration (weekend), hours/day | 8.2 ± 2.2 | - | - | - | 8.2 ± 2.0 |
| Eating habits |  |  |  |  |  |
| Regular | 34 (19.1) | - | - | - | 36 (20.2) |
| A little regular | 58 (32.6) | - | - | - | 63 (35.4) |
| A little irregular | 56 (31.5) | - | - | - | 52 (29.2) |
| Irregular | 30 (16.9) | - | - | - | 27 (15.2) |
| Exercise habits, steps/day | 4636.8 ± 5949.3  (n = 177)^†^ | - | - | - | 6076.7 ± 12132.3  (n = 178) |
| Cognitive impairment (COBRA) |  |  |  |  |  |
| Absence (≤14） | 75 (42.1) | 64 (36.0) | 73 (41.0) | 70 (39.3) | 61 (34.3) |
| Presence (>14） | 103 (57.9) | 114 (64.0) | 105 (59.0) | 108 (60.7) | 117 (65.7) |
| Mean ± SD | 17.3 ± 9.0 | 17.7 ± 8.9 | 17.2 ± 8.9 | 17.6 ± 9.5 | 17.7 ± 8.8 |
| Change from baseline | - | 0.4 ± 5.5  *P =* 0.352^‡^ | −0.1 ± 6.4  *P =* 0.825^‡^ | 0.2 ± 6.9  *P =* 0.632^‡^ | 0.3 ± 6.9  *P =* 0.504^‡^ |
| Depressive symptoms (PHQ-9) |  |  |  |  |  |
| Absence (<10） | 59 (33.1) | 60 (33.7) | 58 (32.6) | 67 (37.6) | 68 (38.2) |
| Presence (≥10） | 119 (66.9) | 118 (66.3) | 120 (67.4) | 111 (62.4) | 110 (61.8) |
| Mean ± SD | 12.7 ± 6.5 | 12.9 ± 6.4 | 12.9 ± 6.8 | 12.8 ± 7.1 | 12.6 ± 7.0 |
| Change from baseline | - | 0.3 ± 4.8  *P =* 0.481^‡^ | 0.2 ± 5.3  *P =* 0.547^‡^ | 0.1 ± 5.8  *P =* 0.846^‡^ | −0.1 ± 6.1  *P =* 0.863^‡^ |
| Sleep disturbance (somnolence) (ESS) |  |  |  |  |  |
| Absence (≤10) | 106 (59.6) | 93 (52.2) | 94 (52.8) | 100 (56.2) | 95 (53.4) |
| Presence (>10) | 72 (40.4) | 85 (47.8) | 84 (47.2) | 78 (43.8) | 83 (46.6) |
| Mean ± SD | 9.8 ± 5.6 | 10.4 ± 5.9 | 10.4 ± 5.9 | 10.2 ± 5.8 | 10.6 ± 5.8 |
| Change from baseline | - | 0.6 ± 3.9  *P =* 0.030^‡^ | 0.6 ± 4.2  *P =* 0.042^‡^ | 0.5 ± 4.3  *P =* 0.151^‡^ | 0.8 ± 4.5  *P =* 0.013^‡^ |
| Sleep disturbance (insomnia) (AIS) |  |  |  |  |  |
| Absence (<10) | 108 (60.7) | 108 (60.7) | 113 (63.5) | 104 (58.4) | 106 (59.6) |
| Presence (≥10) | 70 (39.3) | 70 (39.3) | 65 (36.5) | 74 (41.6) | 72 (40.4) |
| Mean ± SD | 8.2 ± 4.5 | 8.7 ± 4.4 | 8.3 ± 4.5 | 8.7 ± 5.1 | 8.5 ± 4.5 |
| Change from baseline | - | 0.5 ± 3.8  *P =* 0.096^‡^ | 0.1 ± 3.7  *P =* 0.655^‡^ | 0.5 ± 4.2  *P =* 0.093^‡^ | 0.3 ± 4.0  *P =* 0.259^‡^ |
| WPAI-GH (Presenteeism), % | 43.9 ± 29.6  (n = 132) | 45.4 ± 29.4  (n = 127) | 45.8 ± 27.7  (n = 120) | 43.9 ± 29.8  (n = 123) | 43.7 ± 29.9  (n = 126) |
| Change from baseline | - | 2.3 ± 26.7  (n = 116)  *P =* 0.350^‡^ | 4.3 ± 27.9  (n = 110)  *P =* 0.112^‡^ | 0.7 ± 29.2  (n = 110)  *P =* 0.794^‡^ | 2.0 ± 29.7  (n = 107)  *P =* 0.495^‡^ |
| WPAI-GH (Absenteeism), % | 12.5 ± 24.2  (n = 136) | 14.0 ± 25.2  (n = 131) | 14.6 ± 26.0  (n = 126) | 16.2 ± 29.2  (n = 130) | 14.8 ± 28.5  (n = 134) |
| Change from baseline | - | 1.8 ± 26.3  (n = 120)  *P =* 0.464^‡^ | 2.5 ± 30.1  (n = 116)  *P =* 0.380^‡^ | 4.2 ± 32.9  (n = 118)  *P =* 0.172^‡^ | 3.9 ± 33.3  (n = 117)  *P =* 0.211^‡^ |
| WPAI-GH (Overall work impairment), % | 47.6 ± 31.2  (n = 132) | 49.0 ± 30.5  (n = 127) | 49.7 ± 29.0  (n = 120) | 48.8 ± 31.9  (n = 123) | 46.9 ± 31.7  (n = 126) |
| Change from baseline | - | 2.1 ± 27.1  (n = 116)  *P =* 0.403^‡^ | 4.3 ± 29.2  (n = 110)  *P =* 0.129^‡^ | 2.2 ± 32.2  (n = 110)  *P =* 0.480^‡^ | 2.0 ± 32.8  (n = 107)  *P =* 0.532^‡^ |
| WPAI-GH (Activity impairment), % | 46.4 ± 28.8  (n = 137) | 53.8 ± 28.9  (n = 178) | 52.4 ± 29.5  (n = 178) | 51.8 ± 29.6  (n = 178) | 52.5 ± 29.9  (n = 178) |
| Change from baseline | - | 4.7 ± 29.2  (n = 137)  *P =* 0.059^‡^ | 3.9 ± 29.6  (n = 137)  *P =* 0.121^‡^ | 3.1 ± 31.3  (n = 137)  *P =* 0.243^‡^ | 4.3 ± 30.8  (n = 137)  *P =* 0.104^‡^ |
| Indirect cost (Presenteeism), 1000 yen | 1532.3 ± 1095.8  (n = 132) | 1602.4 ± 1138.8  (n = 127) | 1589.1 ± 1013.2  (n = 120) | 1543.1 ± 1125.5  (n = 123) | 1511.5 ± 1050.8  (n = 126) |
| Change from baseline | - | 106.5 ± 1003.8  *P =* 0.256^‡^  (n = 116) | 142.0 ± 994.3  *P =* 0.137^‡^  (n = 110) | 39.3 ± 998.3  *P =* 0.681^‡^(n = 110) | 70.4 ± 1088.3  *P =* 0.505^‡^  (n = 107) |
| Indirect cost (Absenteeism), 1000 yen | 403.0 ± 769.2  (n = 136) | 492.2 ± 886.5  (n = 131) | 522.4 ± 947.6  (n = 126) | 603.8 ± 1156.2  (n = 130) | 518. 4 ± 1029.2  (n = 134) |
| Change from baseline | - | 89.5 ± 927.2  *P =* 0.293^‡^  (n = 120) | 131.6 ± 1085.9  *P =* 0.194^‡^  (n = 116) | 206.1 ± 1277.9  *P =* 0.083^‡^(n = 118) | 154.3 ± 1171.7  *P =* 0.157^‡^  (n = 117) |
| Indirect cost (Overall work impairment), 1000 yen | 1652.7 ± 1135.1  (n = 132) | 1729.3 ± 1176.8  (n = 127) | 1730.9 ± 1067.8  (n = 120) | 1727.4 ± 1242.0  (n = 123) | 1620.6 ± 1120.6  (n = 126) |
| Change from baseline | - | 109.0 ± 1020.9  *P =* 0.253^‡^  (n = 116) | 155.6 ± 1015.3  *P =* 0.111^‡^  (n = 110) | 114.2 ± 1146.4  *P =* 0.299^‡^  (n = 110) | 77.9 ± 1178.6  *P =* 0.496^‡^  (n = 107) |
| QOL (HUI3) | 0.467 ± 0.251 | 0.446 ± 0.239 | 0.459 ± 0.270 | 0.455 ± 0.273 | 0.466 ± 0.260 |
| Change from baseline | - | −0.021 ± 0.196  *P =* 0.151^‡^ | −0.008 ± 0.204  *P =* 0.591^‡^ | −0.012 ± 0.207  *P =* 0.437^‡^ | −0.001 ± 0.216  *P =* 0.961^‡^ |

Data are presented as n (%) or mean ± SD

^†^n = 177 instead of 178 due to the exclusion of an outlier in exercise habits (step count per day).

^‡^Significance versus baseline, paired Student’s t-test.

AIS, Athens Insomnia Scale; COBRA, Cognitive Complaints in Bipolar Disorder Rating Assessment; ESS, Epworth Sleepiness Scale; HUI3, Health Utilities Index Mark 3; PHQ-9, Patient Health Questionnaire-9; QOL, quality of life; SD, standard deviation; WPAI-GH, Work Productivity and Activity Impairment: General Health

**Table S2.** Percent transition of depressive symptoms from baseline to each visit (N = 179)

|  | **Week 12** | | **Week 24** | | **Week 36** | | **Week 48** | |
| --- | --- | --- | --- | --- | --- | --- | --- | --- |
| **Depression** | **Presence** | **Absence** | **Presence** | **Absence** | **Presence** | **Absence** | **Presence** | **Absence** |
| Baseline presence  n = 120 | 103 (57.5) | 17 (9.5) | 104 (58.1) | 16 (8.9) | 99 (55.3) | 21 (11.7) | 93 (52.0) | 27 (15.1) |
| Baseline absence  n = 59 | 16 (8.9) | 43 (24.0) | 17 (9.5) | 42 (23.5) | 13 (7.3) | 46 (25.7) | 18 (10.1) | 41 (22.9) |

Data are presented as n (%). The percentages were calculated using 179 as the denominator.

PHQ-9 score ≥10 (presence) / <10 (absence).

PHQ-9, Patient Health Questionnaire-9

**Table S3.** Correlations between symptom scores

|  | **Week 12**  **(N = 179)** | | **Week 24**  **(N = 179)** | | **Week 36**  **(N = 179)** | | **Week 48**  **(N = 179)** | |
| --- | --- | --- | --- | --- | --- | --- | --- | --- |
|  | ***R*** | ***P*** | ***R*** | ***P*** | ***R*** | ***P*** | ***R*** | ***P*** |
| ΔCOBRA vs ΔPHQ-9 | 0.348 | <0.001 | 0.423 | <0.001 | 0.443 | <0.001 | 0.364 | <0.001 |
| ΔCOBRA vs ΔAIS | 0.062 | 0.409 | 0.269 | <0.001 | 0.337 | <0.001 | 0.337 | <0.001 |
| ΔPHQ-9 vs ΔAIS | 0.418 | <0.001 | 0.476 | <0.001 | 0.573 | <0.001 | 0.506 | <0.001 |
| ΔCOBRA vs ΔESS | 0.284 | <0.001 | 0.315 | <0.001 | 0.277 | <0.001 | 0.419 | <0.001 |

Δ indicates change from baseline.

AIS, Athens Insomnia Scale; COBRA, Cognitive Complaints in Bipolar Disorder Rating Assessment; ESS, Epworth Sleepiness Scale; PHQ-9, Patient Health Questionnaire-9; *R*, Pearson correlation coefficient

**Table S4.** Correlation of work productivity loss with COBRA, PHQ-9, and AIS

|  | **Week 12** | | | **Week 24** | | | **Week 36** | | | **Week 48** | | |
| --- | --- | --- | --- | --- | --- | --- | --- | --- | --- | --- | --- | --- |
|  | **n** | ***R*** | ***P*** | **n** | ***R*** | ***P*** | **n** | ***R*** | ***P*** | **n** | ***R*** | ***P*** |
| Correlation with ΔCOBRA |  |  |  |  |  |  |  |  |  |  |  |  |
| vs ΔPresenteeism | 117 | 0.071 | 0.445 | 111 | 0.029 | 0.761 | 111 | 0.285 | 0.002 | 107 | 0.304 | 0.001 |
| vs ΔAbsenteeism | 121 | 0.015 | 0.869 | 117 | −0.079 | 0.400 | 119 | 0.088 | 0.343 | 117 | 0.095 | 0.310 |
| vs ΔOverall work impairment | 117 | 0.069 | 0.458 | 111 | 0.027 | 0.777 | 111 | 0.250 | 0.008 | 107 | 0.264 | 0.006 |
| vs ΔActivity impairment | 138 | 0.220 | 0.010 | 138 | 0.206 | 0.015 | 138 | 0.303 | <0.001 | 138 | 0.195 | 0.022 |
| Correlation with ΔPHQ-9 |  |  |  |  |  |  |  |  |  |  |  |  |
| vs ΔPresenteeism | 117 | 0.198 | 0.032 | 111 | 0.067 | 0.488 | 111 | 0.295 | 0.002 | 107 | 0.492 | <0.001 |
| vs ΔAbsenteeism | 121 | 0.234 | 0.010 | 117 | 0.169 | 0.068 | 119 | 0.196 | 0.033 | 117 | 0.339 | <0.001 |
| vs ΔOverall work impairment | 117 | 0.280 | 0.002 | 111 | 0.118 | 0.219 | 111 | 0.355 | <0.001 | 107 | 0.507 | <0.001 |
| vs ΔActivity impairment | 138 | 0.228 | 0.007 | 138 | 0.266 | 0.002 | 138 | 0.421 | <0.001 | 138 | 0.398 | <0.001 |
| Correlation with ΔAIS |  |  |  |  |  |  |  |  |  |  |  |  |
| vs ΔPresenteeism | 117 | 0.195 | 0.035 | 111 | 0.174 | 0.068 | 111 | 0.216 | 0.023 | 107 | 0.298 | 0.002 |
| vs ΔAbsenteeism | 121 | 0.313 | <0.001 | 117 | 0.190 | 0.041 | 119 | 0.268 | 0.003 | 117 | 0.234 | 0.011 |
| vs ΔOverall work impairment | 117 | 0.245 | 0.008 | 111 | 0.218 | 0.022 | 111 | 0.270 | 0.004 | 107 | 0.307 | 0.001 |
| vs ΔActivity impairment | 138 | 0.236 | 0.005 | 138 | 0.406 | <0.001 | 138 | 0.355 | <0.001 | 138 | 0.261 | 0.002 |

Δ indicates change from baseline.

AIS, Athens Insomnia Scale; COBRA, Cognitive Complaints in Bipolar Disorder Rating Assessment; PHQ-9, Patient Health Questionnaire-9; *R*, Pearson correlation coefficient

**Table S5.** Single regression analysis: change from baseline to Week 48 in WPAI-GH

|  | **Presenteeism** | | | **Absenteeism** | | | **Overall work impairment** | | | **Activity impairment** | | |
| --- | --- | --- | --- | --- | --- | --- | --- | --- | --- | --- | --- | --- |
| **Explanatory variable** | **Regression coefficient** | **95% CI** | ***P* value** | **Regression coefficient** | **95% CI** | ***P* value** | **Regression coefficient** | **95% CI** | ***P* value** | **Regression coefficient** | **95% CI** | ***P* value** |
| Cognitive impairment (COBRA)  Absence [ref] / Presence | 1.63 | −9.78, 13.04 | 0.777 | −0.10 | −12.87, 12.67 | 0.988 | 1.41 | −11.30, 14.12 | 0.826 | −4.89 | −15.47, 5.69 | 0.362 |
| Depressive symptoms  (PHQ-9)  Absence [ref] / Presence | 1.96 | −9.55, 13.47 | 0.736 | −2.47 | −15.48, 10.55 | 0.708 | −1.09 | −13.92, 11.73 | 0.866 | −0.33 | −11.23, 10.58 | 0.953 |
| Sleep disturbance (somnolence) (ESS)  Absence [ref] / Presence | 4.82 | −6.82, 16.47 | 0.413 | 10.61 | −2.21, 23.44 | 0.104 | 4.88 | −8.11, 17.85 | 0.458 | 6.09 | −4.62, 16.80 | 0.263 |
| Sleep disturbance (insomnia) (AIS)  Absence [ref] / Presence | −1.14 | −13.07, 10.80 | 0.851 | −3.13 | −16.37, 10.12 | 0.641 | −3.98 | −17.26, 9.29 | 0.553 | −5.78 | −16.53, 4.96 | 0.289 |
| Sex  Male [ref] / Female | 0.05 | −11.69, 11.79 | 0.993 | −0.82 | −13.90, 12.25 | 0.901 | −1.37 | −14.44, 11.70 | 0.836 | −11.55 | −22.31, −0.79 | 0.036 |
| Comorbidity  Absence [ref] / Presence | 7.45 | −4.40, 19.30 | 0.215 | 11.36 | −1.59, 24.31 | 0.085 | 7.87 | −5.34, 21.07 | 0.240 | 0.15 | −10.56, 10.86 | 0.978 |
| Highest level of education  Did not graduate university [ref] / University graduate or higher | −6.73 | −18.21, 4.74 | 0.247 | −2.24 | −15.21, 10.74 | 0.733 | −8.56 | −21.32, 4.20 | 0.186 | 0.05 | −10.78, 10.88 | 0.992 |
| Marital status  Unmarried [ref] / Married | −7.93 | −19.51, 3.65 | 0.177 | 6.03 | −6.90, 18.96 | 0.357 | −3.58 | −16.57, 9.42 | 0.586 | −4.41 | −15.21, 6.39 | 0.421 |
| Living with partner  Living alone [ref] / Not living alone | −0.39 | −13.32, 12.55 | 0.953 | 4.59 | −9.96, 19.14 | 0.533 | 0.68 | −13.73, 15.08 | 0.926 | −6.09 | −17.91, 5.74 | 0.310 |
| Alcohol use  None [ref] / ≤ once/month | −4.84 | −19.90, 10.23 | 0.526 | −3.98 | −20.81, 12.85 | 0.640 | −5.85 | −22.30, 10.61 | 0.482 | −1.19 | −15.54, 13.16 | 0.870 |
| None [ref] / 2–4 times/month | −4.01 | −18.90, 10.89 | 0.595 | 3.28 | −13.05, 19.62 | 0.691 | −4.64 | −20.91, 11.63 | 0.573 | −1.61 | −15.82, 12.61 | 0.824 |
| None [ref] / 2–3 times/week | −10.74 | −33.20, 11.73 | 0.345 | −5.95 | −31.87, 19.97 | 0.650 | −12.35 | −36.89, 12.20 | 0.321 | −7.42 | −28.80, 13.96 | 0.494 |
| None [ref] / ≥4 times/week | 15.65 | −5.78, 37.09 | 0.150 | 26.29 | 2.59, 49.98 | 0.030 | 26.08 | 2.66, 49.49 | 0.029 | 8.92 | −9.35, 27.18 | 0.336 |
| Smoking history  Never smoker [ref] / Current smoker | 1.80 | −11.14, 14.74 | 0.783 | 4.31 | −9.69, 18.31 | 0.543 | 2.58 | −11.76, 16.91 | 0.722 | 4.17 | −7.61, 15.96 | 0.485 |
| Never smoker [ref] / Prior smoker | −6.48 | −24.16, 11.20 | 0.469 | −14.35 | −34.76, 6.06 | 0.166 | −11.38 | −30.97, 8.22 | 0.252 | −10.08 | −27.12, 6.97 | 0.244 |
| Eating habits  Regular [ref] / A little regular | 8.41 | −7.41, 24.23 | 0.294 | −9.91 | −27.12, 7.31 | 0.257 | 5.59 | −11.98, 23.15 | 0.529 | 11.19 | −3.45, 25.82 | 0.133 |
| Regular [ref] / A little irregular | 2.94 | −13.46, 19.33 | 0.723 | −3.23 | −21.10, 14.64 | 0.721 | 2.17 | −16.03, 20.38 | 0.813 | −1.59 | −16.49, 13.31 | 0.833 |
| Regular [ref] / Irregular | −5.27 | −26.86, 16.32 | 0.629 | −32.14 | −55.70, −8.57 | 0.008 | −13.50 | −37.47, 10.47 | 0.266 | 3.52 | −14.77, 21.81 | 0.704 |
| Age, years | 0.14 | −0.43, 0.71 | 0.633 | 0.42 | −0.21, 1.05 | 0.191 | 0.23 | −0.40, 0.86 | 0.475 | 0.42 | −0.09, 0.93 | 0.103 |
| Duration of current employment, years | −0.74 | −1.46, −0.01 | 0.047 | 0.25 | −0.61, 1.10 | 0.567 | −0.56 | −1.38, 0.25 | 0.175 | −0.20 | −0.85, 0.45 | 0.541 |
| Duration of disease, years | 0.32 | −0.50, 1.13 | 0.444 | −0.06 | −0.99, 0.86 | 0.893 | 0.31 | −0.60, 1.22 | 0.501 | 0.31 | −0.43, 1.06 | 0.408 |
| Age of diagnosis, years | −0.02 | −0.66, 0.62 | 0.949 | 0.55 | −0.15, 1.25 | 0.121 | 0.10 | −0.62, 0.82 | 0.784 | 0.33 | −0.23, 0.90 | 0.244 |
| Sleep duration (weekdays), hours/day | 3.82 | −0.30, 7.93 | 0.069 | 4.15 | −0.43, 8.73 | 0.075 | 3.79 | −0.80, 8.39 | 0.105 | 1.18 | −2.37, 4.74 | 0.512 |
| Sleep duration (weekends), hours/day | 2.41 | −0.15, 4.96 | 0.064 | 1.33 | −1.59, 4.26 | 0.368 | 2.49 | −0.37, 5.34 | 0.087 | −0.23 | −2.46, 2.01 | 0.842 |
| Exercise habits, steps/day | 0.00 | −0.00, 0.00 | 0.826 | 0.00 | 0.00, 0.00 | 0.313 | 0.00 | −0.00, 0.00 | 0.560 | 0.00 | −0.00, 0.00 | 0.983 |
| Change from baseline to Week 48 in cognitive impairment (COBRA) | 1.20 | 0.31, 2.10 | 0.009 | 0.51 | −0.50, 1.51 | 0.318 | 1.14 | 0.13, 2.15 | 0.028 | 0.93 | 0.09, 1.77 | 0.031 |
| Change from baseline to Week 48 in depressive symptoms (PHQ-9) | 2.50 | 1.59, 3.42 | <0.001 | 2.25 | 1.15, 3.34 | <0.001 | 2.90 | 1.90, 3.91 | <0.001 | 2.21 | 1.29, 3.13 | <0.001 |
| Change from baseline to Week 48 in sleep disturbance (somnolence) (ESS) | 1.20 | −0.20, 2.61 | 0.093 | 0.59 | −0.99, 2.16 | 0.462 | 1.21 | −0.36, 2.78 | 0.130 | 0.33 | −0.93, 1.58 | 0.606 |
| Change from baseline to Week 48 in sleep disturbance (insomnia) (AIS) | 2.30 | 0.77, 3.83 | 0.004 | 2.21 | 0.53, 3.89 | 0.011 | 2.64 | 0.94, 4.34 | 0.003 | 2.42 | 1.03, 3.80 | <0.001 |
| Change in living with partner  Living alone to Living alone [ref] / Living alone to Not living alone | −34.40 | −76.37, 7.57 | 0.107 | −19.03 | −68.77, 30.71 | 0.450 | −41.99 | −88.66, 4.67 | 0.077 | −40.88 | −84.59, 2.83 | 0.067 |
| Living alone to Living alone [ref] / Not living alone to Living alone | −16.40 | −44.38, 11.58 | 0.248 | −2.13 | −35.18, 30.91 | 0.898 | −16.25 | −47.36, 14.86 | 0.303 | −6.60 | −31.53, 18.34 | 0.602 |
| Living alone to Living alone [ref] / Not living alone to Not living alone | −1.97 | −15.28, 11.34 | 0.769 | 3.62 | −11.53, 18.78 | 0.637 | −1.45 | −16.24, 13.35 | 0.847 | −8.50 | −20.63, 3.63 | 0.168 |
| Change in eating habits  Regular to Regular [ref] / Regular to Irregular | 3.11 | −18.66, 24.89 | 0.777 | 6.92 | −16.30, 30.15 | 0.556 | 5.66 | −18.62, 29.93 | 0.645 | −0.79 | −22.13, 20.55 | 0.941 |
| Regular to Regular [ref] / Irregular to Regular | −15.64 | −34.01, 2.73 | 0.094 | −12.34 | −33.17, 8.50 | 0.243 | −16.39 | −36.88, 4.09 | 0.115 | −19.34 | −36.10, −2.57 | 0.024 |
| Regular to Regular [ref] / Irregular to Irregular | 0.09 | −12.95, 13.13 | 0.989 | 0.59 | −14.15, 15.33 | 0.937 | −0.26 | −14.80, 14.28 | 0.972 | −2.58 | −14.42, 9.27 | 0.668 |
| Change in sleep duration (weekdays), hours/day | −1.98 | −6.28, 2.31 | 0.362 | −2.79 | −6.67, 1.10 | 0.159 | −1.21 | −6.00, 3.59 | 0.619 | −1.52 | −5.08, 2.03 | 0.398 |
| Change in sleep duration (weekends), hours/day | −2.71 | −5.60, 0.19 | 0.066 | −1.30 | −4.62, 2.02 | 0.438 | −2.24 | −5.49, 1.01 | 0.174 | −1.89 | −4.37, 0.60 | 0.135 |
| Change in exercise habits, steps/day | 0.00 | 0.00, 0.00 | 0.530 | 0.00 | 0.00, 0.00 | 0.053 | 0.00 | 0.00, 0.00 | 0.138 | 0.00 | 0.00, 0.00 | 0.843 |

AIS, Athens Insomnia Scale; CI, confidence interval; COBRA, Cognitive Complaints in Bipolar Disorder Rating Assessment; ESS, Epworth Sleepiness Scale; PHQ-9, Patient Health Questionnaire-9; ref, reference; WPAI-GH, Work Productivity and Activity Impairment: General Health

**Table S6.** Multiple regression analysis: change from baseline to Week 48 in WPAI-GH

|  | **Presenteeism** | | **Absenteeism** | | **Overall work impairment** | | **Activity impairment** | |
| --- | --- | --- | --- | --- | --- | --- | --- | --- |
|  | ***R*^2^ = 0.302** | | ***R^2^* = 0.136** | | ***R*^2^ = 0.287** | | ***R*^2^ = 0.176** | |
| **Explanatory variable** | **Regression coefficient**  **(95% CI)** | ***P* value** | **Regression coefficient**  **(95% CI)** | ***P* value** | **Regression coefficient**  **(95% CI)** | ***P* value** | **Regression coefficient**  **(95% CI)** | ***P* value** |
| Duration of current employment, years | −0.81  (−1.44, −0.18) | 0.013 | 0.09  (−0.72, 0.90) | 0.827 | −0.67  (−1.38, 0.05) | 0.067 | −0.30  (−0.89, 0.30) | 0.328 |
| Change in cognitive impairment (COBRA) | 0.68  (−0.16, 1.51) | 0.111 | −0.07  (−1.09, 0.95) | 0.893 | 0.54  (−0.40, 1.48) | 0.255 | 0.41  (−0.41, 1.24) | 0.325 |
| Change in depressive symptoms (PHQ-9) | 2.43  (1.38, 3.47) | <0.001 | 2.00  (0.70, 3.30) | 0.003 | 2.81  (1.63, 3.98) | <0.001 | 1.79  (0.74, 2.84) | 0.001 |
| Change in sleep disturbance (insomnia) (AIS) | 0.02  (−1.62, 1.66) | 0.980 | 0.74  (−1.24, 2.72) | 0.460 | 0.12  (−1.73, 1.97) | 0.894 | 1.02  (−0.53, 2.57) | 0.195 |

AIS, Athens Insomnia Scale; CI, confidence interval; COBRA, Cognitive Complaints in Bipolar Disorder Rating Assessment; PHQ-9, Patient Health Questionnaire-9; *R^2^*, coefficient of determination; WPAI-GH, Work Productivity and Activity Impairment Questionnaire: General Health

**Table S7.** Single regression analysis: change from baseline to Week 48 in QOL (HUI3)

| **Explanatory variable** | **Regression coefficient** | **95% CI** | ***P* value** |
| --- | --- | --- | --- |
| Cognitive impairment (COBRA)  Absence [ref] / Presence | 0.04 | −0.02, 0.11 | 0.189 |
| Depressive symptoms (PHQ-9)  Absence [ref] / Presence | 0.09 | 0.02, 0.16 | 0.010 |
| Sleep disturbance (somnolence) (ESS)  Absence [ref] / Presence | −0.00 | −0.07, 0.06 | 0.900 |
| Sleep disturbance (insomnia) (AIS)  Absence [ref] / Presence | 0.13 | 0.06, 0.19 | <0.001 |
| Sex  Male [ref] / Female | −0.02 | −0.09, 0.05 | 0.543 |
| Comorbidity  Absence [ref] / Presence | −0.04 | −0.10, 0.03 | 0.262 |
| Highest level of education  Did not graduate university [ref]] / University graduate or higher | −0.00 | −0.07, 0.06 | 0.926 |
| Marital status  Unmarried [ref] / Married | −0.02 | −0.09, 0.04 | 0.517 |
| Living with partner  Living alone [ref] / Not living alone | 0.02 | −0.06, 0.09 | 0.685 |
| Alcohol use  None [ref] / ≤once/month | 0.11 | 0.03, 0.20 | 0.009 |
| None [ref] / 2–4 times/month | −0.01 | −0.10, 0.08 | 0.802 |
| None [ref] / 2–3 times/week | 0.07 | −0.06, 0.19 | 0.286 |
| None [ref] / ≥4 times/week | −0.04 | −0.15, 0.06 | 0.405 |
| Smoking history  Never smoker [ref] / Current smoker | −0.02 | −0.09, 0.05 | 0.625 |
| Never smoker [ref] / Prior smoker | 0.02 | −0.08, 0.12 | 0.673 |
| Eating habits  Regular [ref] / A little regular | 0.03 | −0.07, 0.12 | 0.551 |
| Regular [ref] / A little irregular | −0.00 | −0.10, 0.09 | 0.928 |
| Regular [ref] / Irregular | 0.04 | −0.07, 0.14 | 0.508 |
| Age, years | −0.00 | −0.01, 0.00 | 0.228 |
| Duration of current employment, years | 0.00 | −0.00, 0.00 | 0.927 |
| Duration of disease, years | −0.00 | −0.01, 0.00 | 0.304 |
| Age at diagnosis, years | −0.00 | −0.00, 0.00 | 0.573 |
| Sleep duration (weekdays), hours/day | −0.01 | −0.03, 0.02 | 0.557 |
| Sleep duration (weekends), hours/day | −0.00 | −0.02, 0.01 | 0.940 |
| Exercise habits, steps/day | 0.00 | 0.00, 0.00 | 0.883 |
| Work productivity (WPAI-GH) |  |  |  |
| Presenteeism | 0.00 | 0.00, 0.00 | 0.118 |
| Absenteeism | 0.00 | 0.00, 0.00 | 0.164 |
| Overall work impairment | 0.00 | 0.00, 0.00 | 0.222 |
| Activity impairment | 0.00 | −0.00, 0.00 | 0.269 |
| Change from baseline to Week 48 in cognitive impairment (COBRA) | −0.01 | −0.01, −0.01 | <0.001 |
| Change from baseline to Week 48 in depressive symptoms (PHQ-9) | −0.02 | −0.02, −0.01 | <0.001 |
| Change from baseline to Week 48 in sleep disturbance (somnolence) (ESS) | −0.01 | −0.01, 0.00 | 0.100 |
| Change from baseline to Week 48 in sleep disturbance (insomnia) (AIS) | −0.02 | −0.03, −0.02 | <0.001 |
| Change in living with partner  Living alone to Living alone [ref] / Living alone to Not living alone | 0.01 | −0.24, 0.26 | 0.912 |
| Living alone to Living alone [ref] / Not living alone to Living alone | 0.15 | −0.00, 0.30 | 0.053 |
| Living alone to Living alone [ref] / Not living alone to Not living alone | 0.01 | −0.07, 0.08 | 0.894 |
| Change in eating habits  Regular to Regular [ref] / Regular to Irregular | −0.03 | −0.15, 0.10 | 0.685 |
| Regular to Regular [ref] / Irregular to Regular | 0.04 | −0.06, 0.14 | 0.466 |
| Regular to Regular [ref] / Irregular to Irregular | −0.03 | −0.10, 0.04 | 0.400 |
| Change in sleep duration (weekdays), hours/day | 0.01 | −0.01, 0.02 | 0.470 |
| Change in sleep duration (weekends), hours/day | 0.00 | −0.01, 0.02 | 0.891 |
| Change in exercise habits, steps/day | 0.00 | 0.00, 0.00 | 0.207 |
| Change from baseline to Week 48 in work productivity (WPAI-GH) |  |  |  |
| Presenteeism | −0.00 | −0.00, −0.00 | 0.006 |
| Absenteeism | −0.00 | −0.00, 0.00 | 0.017 |
| Overall work impairment | −0.00 | −0.00, −0.00 | 0.005 |
| Activity impairment | −0.00 | −0.00, −0.00 | 0.001 |

AIS, Athens Insomnia Scale; CI, confidence interval; COBRA, Cognitive Complaints in Bipolar Disorder Rating Assessment; ESS, Epworth Sleepiness Scale; HUI3, Health Utilities Index Mark 3; PHQ-9, Patient Health Questionnaire-9; QOL, quality of life; ref, reference; WPAI-GH, Work Productivity and Activity Impairment Questionnaire: General Health
